# Current Treatment Options in CLL

**DOI:** 10.3390/cancers13102468

**Published:** 2021-05-19

**Authors:** Moritz Bewarder, Stephan Stilgenbauer, Lorenz Thurner, Dominic Kaddu-Mulindwa

**Affiliations:** Department of Hematology, Oncology, Clinical Immunology, Rheumatology, Medical School, University of Saarland, 66424 Homburg, Germany; moritz.bewarder@uks.eu (M.B.); stephan.stilgenbauer@uks.eu (S.S.); lorenz.thurner@uks.eu (L.T.)

**Keywords:** chronic lymphocytic leukemia, BTK inhibitor, BCL2 inhibitors, treatment

## Abstract

**Simple Summary:**

Standard treatment for chronic lymphocytic leukemia (CLL) has experienced a dramatic change over the last years. Until recently, CLL was treated using chemotherapy in combination with anti-CD20 antibody-based immunotherapy. Depending on age and clinical condition, patients received more or less intensive chemotherapy and were at risk of side effects commonly associated with chemotherapy. Currently, patients are mostly treated with so-called novel agents, including BTK inhibitors, Bcl-2 inhibitors and PI3K inhibitors, which are generally well tolerated but have a specific side effect profile. CLL is a chronic disease; therefore, most patients will relapse on or after treatment with these drugs and will require multiple lines of therapy. In this review, we present the current treatment options for patients with CLL and discuss the optimal treatment approaches and sequences, taking into account the specific side effects of each novel agent in the context of different clinical settings.

**Abstract:**

After impressive developments in recent years with the rise of new targeted agents, chemoimmunotherapy (CIT) only plays a minor role in the treatment of patients with chronic lymphocytic leukemia (CLL). Inhibitors of the Bruton tyrosine kinase (BTK), such as ibrutinib or more recently acalabrutinib, are highly effective, even in poor-risk or chemo-refractory patients. Venetoclax, an inhibitor of the anti-apoptotic BCL2 protein and, to a lesser extent, phosphoinositide-3 kinase (PI3K) delta inhibitors, add to the armamentarium of targeted agents for the treatment of CLL. Furthermore, anti-CD20 monoclonal antibodies are used very successfully either alone or in combination with BTK, BCL2 or PI3K inhibitors. Despite these advances, there is still an ongoing pursuit for new therapeutic approaches in the treatment of CLL. An even bigger challenge poses the determination of the optimal combination and sequence of those drugs. Here, we give an overview of current treatment options in CLL, weighing the advantages and disadvantages of each approach in the light of different clinical settings.

## 1. Introduction

In the Western world, chronic lymphocytic leukemia (CLL) remains the most common leukemia in adults [1,2], with an average age of approximately 70 years at the time of diagnosis [1,3]. Its incidence is 4.2/100,000 population per year and rises to over 30/100,000 in people >80 years of age. Nevertheless, routine screening for CLL is not recommended at any age [3]. Diagnostic criteria for CLL are assessed by blood smear and immunophenotyping, requiring the presence of ≥5 × 10^9^/L monoclonal B lymphocytes in the peripheral blood, sustained for at least 3 months with a specific immunophenotype co-expressing CD5, CD19, and CD23 [4]. Clonal disease is determined by light chain restriction assessed by flow cytometry. Malignant cells are morphologically mature lymphocytes with sparse cytoplasm and condensed nuclei. Prolymphocytes with prominent nucleoli constitute fewer than 55% of lymphoid cells [5]. CLL has a heterogenous clinical course which is mostly indolent, but can be more aggressive with rapid progression in some cases [4]. It is thought that underlying genetic alterations are mainly responsible for individual disease courses, with the most relevant genetic aberrations being del(17p), TP53-mutation, and unmutated IGHV status [6,7,8,9,10]. The CLL International Prognostic Index (CLL-IPI), which combines genetic, biochemical, and clinical parameters, can be used as a prognostic tool before the initiation of treatment [11]. It includes TP53-, IGHV-mutational status, serum β2-microglobulin concentration, clinical stage, and age, and allows physicians to take a more targeted approach to the management of patients with CLL. Although well established in the setting of chemoimmunotherapy (CIT), its role in the era of front-line treatment with targeted agents is yet to be determined [12]. Over the last few years, there have been tremendous efforts to improve the treatment for patients with CLL, resulting in the development of targeted therapies trying to replace classic cytostatic agents. Despite these improvements, allogeneic stem cell therapy still remains the only curative treatment option [13]. Chemoimmunotherapy with fludarabine, cyclophosphamide, and rituximab (FCR) has been the standard of care for young, fit patients [14,15,16], even though it is limited by its side effects and reduced activity in patients with genetic risk factors such as TP53 mutation, del(17p), del(11q), NOTCH1 mutation and unmutated IGHV status [8]. With the introduction of the Bruton tyrosine kinase inhibitor (BTKi) ibrutinib, which irreversibly inhibits Bruton tyrosine kinase (BTK), an essential enzyme in the B cell receptor (BCR) signaling pathway, the era of targeted agents for CLL patients began [17,18,19]. Recently, acalabrutinib, a second-generation BTKi with higher selectivity for BTK than ibrutinib [20], was approved by the U.S. Food and Drug Administration (FDA) and the European Medicines Agency (EMA) for the treatment of CLL patients.

Shortly after the approval of ibrutinib, the combination of idelalisib ± rituximab, a phosphoinositide 3-kinase (PI3K) delta inhibitor, found its way into the European Society for Medical Oncology (ESMO) clinical practice guidelines for CLL in 2015 [21,22]. In 2018, another PI3K inhibitor, duvelisib, was approved for relapsed/refractory (r/r) CLL by the FDA [23]. Moreover, with the novel agent venetoclax, which inhibits anti-apoptotic BCL2, leading to the apoptosis of CLL cells and is used in different clinical settings from first-line to relapse settings [24,25,26,27], physicians now have a broad spectrum of treatment options. Despite all these improvements, treatment is only initiated when patients develop active disease according to the criteria of the International Workshop on Chronic Lymphocytic Leukemia [4] (iwCLL), and a “watch and wait” approach is feasible in many cases.

The purpose of this review is to provide an overview of current treatment options in CLL with high relevance in clinical practice.

## 2. Watch and Wait

When CLL is diagnosed at an early disease stage, as determined according to Rai or Binet [28] (Binet A and B or Rai 0, I and II without active disease [29,30]), no therapy or risk assessment is necessary and patients should be monitored every 3 months in the first year and disease dynamic-adapted thereafter [3]. This “watch and wait” approach is justified because early treatment with chemotherapy (chlorambucil or fludarabine) does not result in prolonged overall survival [31,32]. Whether early treatment with the BTK inhibitor ibrutinib results in prolonged overall survival (OS) is currently being investigated by the German CLL Study Group in the CLL12 trial [33].

## 3. Chemoimmunotherapy

Even though novel agents such as BTKi and BCL2 inhibitors are the standard of care in most therapeutic settings, chemoimmunotherapy (CIT) still has its place in the treatment landscape of CLL. The combination of the chimeric CD20 antibody rituximab with fludarabine and cyclophosphamide (FCR regime) has been the standard for young, fit patients for more than a decade. Rituximab, which is used for most B cell malignancies, activates the classical pathway of the complement system and triggers antibody-dependent cell-mediated cytotoxicity (ADCC) [34]. The FCR regime was established by the CLL-8 trial, a phase 3 randomized, controlled trial, which included young, fit (CIRS score < 6 and glomerular filtration rate > 70 mL/min) patients with CLL (BINET B or C) [15]. It compared FCR and FC (fludarabine, cyclophosphamide), and showed an improvement in PFS for FCR (56.8 months versus 32.9 months, hazard ratio (HR) 0.59; *p* < 0.001), while the median overall survival (OS) was not reached in the FCR arm versus 86 months in the FC arm. There are certain cytogenetic/molecular subgroups which benefit the most from FCR, i.e., patients with immunoglobulin heavy chain variable region (IGHV) mutated CLL whos median PFS has not been reached [16]. These data indicate that there might be a proportion of young, fit patients with IGVH-mutated CLL that could potentially be cured with FCR [35]. In contrast, patients with unmutated IGHV, mutated TP53, mutated NOTCH1, del(17p), del(11q) showed inferior PFS. Therefore, it is crucial to identify the subgroup of patients who might benefit from FCR treatment achieving long-term disease control. The side effects of FCR, such as hematological toxicity and infectious complications, are well documented [36,37], making it a feasible treatment choice only for young, fit patients because dose reductions in FCR, which are an often used approach to minimize the drug-related toxicity of FCR, result in reduced efficacy [38,39]. Although FCR is still a reasonable treatment option for a small proportion of CLL patients, recent trials such as the CLL-13 trial by the German CLL Study Group (GCLLSG) [40] or the FLAIR trial [41] are challenging CIT and are probably shifting the standard of care to targeted agents even more. In addition, FCR had a higher mortality than expected in recent phase 3 trials [42].

For elderly patients with CLL, the bendamustine–rituximab (BR) combination rather than FCR is preferred if CIT is the treatment of choice. The subgroup analysis of patients >65 years old in the CLL-10 trial of the GCLLSG (FCR vs. BR) showed a better toxicity profile, with increased OS in this patient group for the BR regime (78.8% vs. 70.9%) [43,44]. Of interest, the MABLE trial indicated superior outcomes with BR in fludarabine-ineligible patients with a lower bendamustine dose of 70mg/m^2^ [45,46]. Even though BR is effective, especially in elderly patients (>65 years) with untreated CLL, it showed inferiority compared to ibrutinib in a direct comparison. The phase 3 Alliance trial by Woyach et al. showed that ibrutinib was superior to treatment with BR with regard to PFS (74% vs. 87%), but without a significant difference between the treatment groups regarding OS [47]. Of note, grade ≥3 non-hematologic adverse events rates were lower with bendamustine plus rituximab (63% vs. 74%), reflecting its still-valuable role in the treatment setting for elderly patients; however, this could also be explained by a temporary versus a permanent therapy.

Taking into account that the median age at diagnosis of CLL is 65–70 years, which makes the occurrence of comorbidities in these patients more likely, there is urgent need for less toxic therapeutic options. For decades, chlorambucil (clb) has been the standard of care for elderly, frail patients, even though, as a single agent, it only showed modest overall response rates (ORR) of 37% with a median PFS of 14 months in previous trials [48]. To improve the response rates, CD20-antibodies were added to chlorambucil as a chemotherapy backbone. The addition of rituximab to chlorambucil led to an improved ORR (84%), with a median PFS of 23.5 months in a phase 2 study [49]. The second CD20 monoclonal antibody which was used as a combination partner for chlorambucil is obinutuzumab (GA101). It is a glycoengineered type II CD20 and immunoglobulin G1 Fc-optimized monoclonal antibody with a superior efficacy due to direct cytotoxicity and enhanced ADCC [50]. Even as monotherapy, it showed a response rate of 62% in heavily pretreated patients [51].

The phase 3 CLL11 trial compared rituximab–chlorambucil with Obinutuzumab–chlorambucil and chlorambucil monotherapy. The combination treatment arms showed better ORR and PFS, with a median PFS of 11.1 (clb), 15.2 (rituximab–clb) and 26.7 (obinutuzumab–clb) months, respectively. In addition, obinutuzumab–clb was more frequently associated with undetectable minimal residual disease (uMRD) [52], and obinutuzumab led to a longer time to next therapy (TTNT) and improved OS, with the median OS not reached for the obinutuzumab–clb group versus 73.1 months in the rituximab–clb arm [53]. However, even though it was used as a comparison in clinical trials for elderly or frail CLL patients over decades, newer, well-tolerated therapeutics such as BTKi or BCL2 inhibitors replaced clb as the standard of care in elderly or frail patients [27,54].

## 4. BCL2 Inhibitors

Increased expression of the antiapoptotic protein B cell lymphoma 2 (BCL2) seems to be responsible for rendering CLL cells resistant to apoptosis [55,56]. ABT-737 (navitoclax) mimics BH3, a physiologic BCL2 antagonist, and was the first drug of this class to be tested in clinical trials [57,58,59]. Initial results were promising, but further use of ABT-737 was prevented by the occurrence of severe thrombocytopenia due to the concurrent inhibition of BCL-xL, a protein required for platelet survival [60]. Venetoclax, formerly known as ABT-199, is an orally administered inhibitor of BCL2 with higher selectivity for BCL2 but less activity against BCL-xL, inducing the apoptosis of CLL cells in a TP53-independent manner [24,61,62]. As a highly effective oral inhibitor of BCL2, venetoclax was first approved in the r/r treatment setting for CLL patients with del(17p), based on the two phase 2 trials M13-928 [25] and M14-032 [63]. Of note, for patients in the M13-982 trial with del(17p), venetoclax showed an ORR of 77% and CR rate of 20% with a high uMRD rate of 27% in peripheral blood, underlining its ability to induce deep remissions in this high-risk CLL subgroup [25,64]. Over time, the initial issue of tumor lysis syndrome (TLS) has been successfully mitigated by the implementation of a dose ramp-up schedule. In detail, the venetoclax dose is raised from 20 mg to 50 mg to 100 mg to 200 mg to 400 mg (target dose) daily in a step-by-step fashion every week [24].

Later on, the phase 3 MURANO trial showed improved PFS for the combination of venetoclax and rituximab (VR) versus BR. In the MURANO trial, venetoclax was administered for 24 cycles (400 mg daily) in combination with rituximab (six cycles every 4 weeks) versus six cycles of CIT (BR) in patients with r/r CLL, including patients previously treated with BTKi-based regimens. The combination of rituximab and venetoclax led to a significant improvement of the 24 month PFS (84.9% vs. 36.3%) [26]. This advantage was seen across all high-risk subgroups, including patients with del(17p) and unmutated IGHV status. In addition, the high ORR (92%) resulted in an impressive uMRD rate of 84% in the peripheral blood [26]. The sustained benefit of VR was shown in the 3-year follow-up analysis, with PFS rates of 71.4% vs. 15.2% for BR and superior survival rates (OS 87.9% vs. 79.5%) [65]. Most recently, with a median follow-up of 59.2 months, the VR group demonstrated a median PFS of 53.6 months compared to 17 months for BR and 5-year OS estimates of 82.1% vs. 62.2% for BR, respectively, confirming the initial results [66]. The PFS estimate 36 months after the end of treatment (EOT) was 51.1% for patients who completed 2 years of venetoclax. Interestingly, for VR patients who reached EOT without disease progression, uMRD at EOT (83/118) predicted improved OS (3-year post-EOT survival estimate 95.3% versus 85% for those [35/118] with detectable MRD at EOT). In the context of venetoclax therapy in r/r CLL patients, peripheral blood MRD assessment has been shown to be a good surrogate for bone marrow MRD assessment, with reliable correlation with long-term outcomes [67].

The approval of venetoclax as a second-line treatment for all CLL patients, regardless of their del(17p) status, was made in June 2018 by the FDA, while the EMA approved the combination of venetoclax and rituximab in October 2018. Both agencies based their decision on the results of the MURANO trial [26].

Moving on, venetoclax recently found its way into the first-line treatment of CLL patients due to the results of the CLL14 trial, a phase 3 trial which investigated the combination of venetoclax and obinutuzumab in mostly elderly patients with comorbidities vs. obinutuzumab–clb [27]. The combination of venetoclax and obinutuzumab led to an improved 24-month PFS (88.2% vs. 64.1%), which was also observed in patients with del(17p), TP53 mutation, or both, as well as in patients with non-mutated IGHV. The recently presented 3-year follow-up showed a high ongoing rate of uMRD for the combination of venetoclax–obinutuzumab vs. obinutuzumab–clb (47.2% vs. 7.4%), emphasizing the potential of venetoclax [68]. Of note, patients in the CLL14 trial were over 70 years old, with a median age of 72 years, and had comorbidities with a median Cumulative Illness Rating Scale score of 8 and a median creatinine clearance of 66.4 ml/minute. The efficacy of venetoclax in younger, fit patients still needs to be proven. This question will probably be answered by the CLL13 trial (NCT02950051), which compares CIT (FCR or BR) vs. various combinations of venetoclax (Ve), rituximab (R), obinutuzumab (G) and ibrutinib (I) (RVe vs. GVe vs. GIVe) in treatment-naïve, fit CLL patients without del(17p) or TP53 mutation [40].

In summary, venetoclax induces high response rates and deep remissions, even in high-risk subgroups with MRD negativity and prolonged PFS, giving physicians a time-limited treatment option [65].

## 5. BTK Inhibitors

### 5.1. Ibrutinib

Ibrutinib, which is an oral inhibitor of BTK, leads to an inhibition of CLL-cell migration, survival and, most importantly, proliferation [69,70], resulting in high ORR when continuously administered. Ibrutinib binds covalently to cysteine 481, which is part of the adenosine triphosphate (ATP)-binding pocket of BTK [71]. It was first approved for the treatment of CLL patients by the FDA in 2014 in the relapsed/refractory setting, based on a phase 1b/2 trial (PCYC-1102) where 85 patients with r/r CLL received a daily dose of 420 or 840 mg of ibrutinib [17]. In this trial, the ORR was 71%, independent of clinical or genomic risk factors in both dose groups [18]. The recently published final analysis of the RESONATE trial with a median follow-up of 65.3 months demonstrated a significantly longer median PFS for r/r CLL patients treated with ibrutinib compared to ofatumumab (44.1 versus 8.1 months, *p* < 0.001) [72]. These results were consistent in all high-risk subgroups of patients (del(17p), TP53 mutation, unmutated IGHV status, del(11q)) who comprised 82% of the entire study population. For high-risk relapsed CLL patients with del(17p) and a median of two prior therapies, the phase 2 RESONATE-17 trial (PCYC-1117) resulted in a PFS at 24 months of 63% [73].

Regarding treatment-naïve (tn) CLL patients, ibrutinib was compared to chlorambucil in elderly patients (≥65 years) in the RESONATE-2 trial, with an improved ORR (ORR; 86% vs. 35%), 2-year PFS (89% vs. 34%), and 2-year OS (98% vs. 85%) [19]. The long-term follow-up showed an estimated 5-year PFS and OS of 70% and 83% for ibrutinib compared to 12% and 68% for chlorambucil, respectively. The investigator-assessed ORR was 92%, with a CR rate of 30% [54]. The trial led to the approval for ibrutinib monotherapy in patients with previously untreated CLL.

The efficacy of ibrutinib was further confirmed in two phase 3 trials. The Alliance trial (A041202) evaluated the efficacy of ibrutinib either alone or in combination with rituximab (ibrutinib ± rituximab, IR) compared to CIT (bendamustine plus rituximab, BR) in the first-line treatment of older CLL patients (≥65 years) [47]. The other trial (E1912) evaluated the efficacy of ibrutinib plus rituximab for six cycles (after an initial cycle of ibrutinib monotherapy), followed by ibrutinib until disease progression, compared to six cycles of CIT with fludarabine, cyclophosphamide, and rituximab (FCR) in patients 70 years of age or younger [74]. Both studies showed an improved PFS for ibrutinib-treated patients. However, the uMRD rate was significantly lower with ibrutinib-containing regimens in both trials [47,74]. CIT with BR resulted in a uMRD rate of 8%, compared to 1% with ibrutinib and 4% with IR, respectively [47]. In addition, the percentage of patients with uMRD with FCR treatment was higher in the E1912 trial as well, as compared to IR (59.2% vs. 8.3%) [74]. It is worth noting that in the E1912 trial, at a median follow-up of 33.6 months, the combination of ibrutinib plus rituximab showed an improved 3-year PFS rate of 89.4% vs.** 72.9% (HR 0.35; 95% CI 0.22–0.56; *p* <  0.001) compared to CIT in patients with unmutated IGHV status. Nevertheless, in patients with IGHV-mutated CLL, there was no significant difference regarding 3-year PFS (87.7% in the IR group vs. 88.0% in the CIT group; HR 0.44; 95% CI 0.14–1.36) [74].

Although extended follow-up of the original RESONATE-2 study suggested that remissions deepen over time with continued ibrutinib therapy, it is clear that continuous and indefinite therapy with ibrutinib is required to maintain clinical benefit [75].

In elderly patients and comorbid patients, the iLLUMINATE trial tested the combination of ibrutinib and obinutuzumab against chlorambucil and obinutuzumab and showed a benefit in PFS for the combination of ibrutinib and obinutuzumab versus chlorambucil and obinutuzumab [76], leading to the FDA and EMA approval of this combination. This phase 3 trial randomized 229 patients with tn CLL aged ≥65 years or younger than 65 years with existing comorbidities to receive six cycles of obinutuzumab in combination with continuous daily ibrutinib (until disease progression) or chlorambucil (0.5 mg/kg on days 1 and 15 every 28 days for six cycles). The median PFS in the ibrutinib and obinutuzumab group had not been reached after a median follow-up of 31.3 months, compared to a median PFS of 19 months in the chlorambucil and obinutuzumab group (*p* < 0.0001). The safety analysis showed a higher AE rate in the ibrutinib and obinutuzumab group (58% serious AEs vs. 35% in the chlorambucil and obinutuzumab group, respectively). The trial did not include an ibrutinib monotherapy arm; therefore, the benefit of adding obinutuzumab to ibrutinib remains unclear.

### 5.2. Acalabrutinib

Acalabrutinib was recently approved by the FDA for tn and r/r CLL. It is a second-generation and more selective irreversible BTK inhibitor [20]. Compared to ibrutinib, acalabrutinib shows equipotent BTK inhibition [77] but without the off-target effects on other kinases [78,79], which probably explains some of the side effects of ibrutinib [80]. Acalabrutinib seems to have less inhibitory effect on healthy T cells compared to ibrutinib, probably due to its higher selectivity [80]. Acalabrutinib was first evaluated in a phase 1/2 trial that included patients with relapsed CLL or small lymphocytic leukemia (SLL) in need of treatment according to the IWCLL guidelines with at least one prior CLL therapy [81]. Adequate organ function, exclusion of active infection, and an ECOG performance score ≤ 2 was required. A total of 61 patients with a median age of 62 years were included; 31% had deletion(17p) and 75% had an unmutated IGHV status. The ORR was 95% with a well-tolerated safety profile, which appeared to be similar to that reported with ibrutinib. Interestingly, in all patients with deletion(17p), a response to acalabrutinib was documented.

Acalabrutinib was then tested in patients with treatment-naïve CLL in a phase 3, multicenter trial (ELEVATE TN) [82]. In this trial, patients had a median age of 70 years, and were randomized to receive acalabrutinib (Acb) with (*n* = 179) or without (*n* = 179) obinutuzumab (G) or chlorambucil (Clb) with obinutuzumab (*n* = 177). It included patients older than 65 years and younger than 65 years with concurrent comorbidities (Cumulative Illness Rating Scale for Geriatrics score > 6 [83] or renal impairment with a creatinine clearance of 30–69 mL/min). Patients with significant cardiovascular disease were excluded, and concomitant treatment with warfarin or equivalent vitamin K antagonists was not allowed. At median follow-up of 28.3 months (IQR 25.6–33.1), median PFS was longer with G-Acb (not reached) and Acb monotherapy (not reached), compared to G-Clb (22.6 months, 20.2–27.6). Estimated PFS at 24 months was 93% with G-Acb (95% CI 87–96%) and 87% with Acb monotherapy (95% CI 81–92%) versus 47% with G-Clb (95% CI 39–55%).

The recently published ASCEND study is a phase 3, multicenter trial which compared acalabrutinib with treatment according to the investigator’s choice (idelalisib plus rituximab or BR) in patients with r/r CLL. Patients had a median age of 67 years (32–90) and had received a median of two therapies prior to study inclusion [84]. In this trial, 16% had a del(17p) with a reported estimated 12-month PFS of 88% for acalabrutinib vs. 68% or 69% for idelalisib plus rituximab or BR, respectively. Cross-over to acalabrutinib (23%) was allowed, and the 12-month OS rates were 94% for patients treated with acalabrutinib vs. 91% for patients treated either with idelalisib plus rituximab or BR. Regarding adverse events, acalabrutinib showed a favorable safety profile when compared to idelalisib plus rituximab, with serious adverse events occurring in 29% (acalabrutinib monotherapy), 56% (idelalisib + rituximab), and 26% (BR) of patients. The adverse events profile was as expected, and treatment discontinuation (11%) or dose reductions (3%) of acalabrutinib were rare.

## 6. PI3K Inhibitors

In 2014, with the approval of the phosphoinositide 3-kinase delta (PI3Kδ) inhibitor idelalisib by the FDA and EMA for patients with r/r CLL, an additional compound became available to physicians for a targeted CLL treatment approach. In relapsed CLL patients with decreased renal function, previous therapy-induced myelosuppression, or major coexisting illnesses, idelalisib, in combination with rituximab, significantly improved ORR, PFS, and OS, as compared to rituximab alone [21]. These findings were also seen in the prespecified subgroups of high-risk patients with del(17p), TP53 mutation, or unmutated IGHV status. When used as first-line therapy in CLL patients, idelalisib lead to severe immune-mediated hepatotoxicity, especially in younger patients with mutated IGHV status. Lymphocytic infiltration of the liver, reduced regulatory T cells, and increased levels of the proinflammatory cytokines CCL-3 and CCL-4 were reported for patients experiencing hepatotoxicity (elevation of transaminases). These findings indicate immune-related mechanisms in the pathogenesis of idelalisib-induced hepatotoxicity [85]. Moreover, many patients developed gastrointestinal symptoms during idelalisib therapy, which is probably the most clinically significant side effect of idelalisib. Weidner et al. assumed that intraepithelial lymphocytosis, epithelial cell apoptosis, and neutrophilic cryptitis are effects of idelalisib leading to gastrointestinal injury [86,87]. In line with this, in vitro studies suggest that PI3Kδ inhibition decreases the activity of regulatory T cells (Tregs), thus stimulating an anti-tumor immune response [88]. Simultaneously to the publication of the study of Lampson et al., Gilead closed seven trials due to toxicity concerns including predominantly infectious complications such as pneumocystis jirovecii pneumonia (PJP) and cytomegalovirus (CMV) reactivation [85]. Therefore, it is now recommended to administer PJP prophylaxis with co-trimoxazole and to monitor for CMV reactivation under treatment with idelalisib [3,4]. Despite these concerns, idelalisib remains an effective treatment option for a small proportion of patients with r/r CLL.

In 2018, the FDA approved the second PI3K inhibitor, duvelisib. It was first developed as IPI-145 and inhibits both the γ- and δ-isoforms of PI3K. In in vitro studies, duvelisib led to the apoptosis of CLL cells using sub-nanomolar concentrations. Inhibition of the δ-isoform of PI3K blocks the survival and proliferation of CLL cells [89,90,91], whereas inhibition of the γ-isoform modulates the microenvironment of malignant cells [92,93,94,95,96,97]. The enhanced activity of duvelisib is thought to be related to the dual inhibition of the PI3K γ- and δ-isoforms [98]. A first phase 1 trial demonstrated a tolerable safety profile and clinical activity for duvelisib in advanced hematologic malignancies [99]. Regarding r/r and tn CLL patients, duvelisib showed an ORR of 56% and 83%, respectively [99]. Compared to idelalisib, the toxicities of duvelisib were similar in this trial, with observed effects of neutropenia (20% grade ≥ 3), hepatotoxicity (19.5% transaminitis grade ≥ 3), late-onset diarrhea/colitis (11%/6% grade ≥ 3), infections (10% grade ≥ 3), including three patients with pneumocystis jirovecii pneumonia and two systemic CMV infections, and interstitial pneumonitis (4%) [99].

The FDA approval of duvelisib was based on the publication of the pivotal phase 3 DUO trial [23]. In this trial, 319 patients with r/r CLL were randomly assigned to receive either duvelisib or ofatumumab. Serious adverse events were of special interest in this trial. Adverse events ≥ grade 3 occurred more often in the duvelisib arm than in patients treated with ofatumumab (87% vs. 48%) including diarrhea, neutropenia, pyrexia, nausea, and anemia. PFS and ORR were significantly improved with duvelisib as compared to ofatumumab (PFS: median, 13.3 vs. 9.9 months; ORR: 74% vs. 45%).

As with idelalisib, the treatment benefit of duvelisib included patients with del(17p) and TP53 mutation. Nevertheless, at present, it has only been approved in the United States by the FDA, and due to the abovementioned side effects, it should be used with caution.

## 7. Investigational Approaches and Perspectives

### Zanubrutinib

Zanubrutinib inhibits BTK irreversibly with similar affinity as ibrutinib, but shows significantly decreased affinity to other kinases that contain a cysteine at the ATP-binding site [100,101]. The relatively high affinity of ibrutinib to other kinases seems to be responsible for its off-target effects that include bleeding, atrial fibrillation, rash, and diarrhea [102,103,104]. Zanubrutinib is much more specific for BTK; therefore, it is thought to be associated with fewer toxicities. Since November 2019, zanubrutinib has been approved by the FDA for the treatment of mantle cell lymphoma after at least one line of therapy, and in February 2021, the FDA accepted the supplemental new drug application for the treatment of patients with Waldenstrom’s macroglobulinemia [105,106].

The initial phase 1 trial investigating the safety and efficacy of zanubrutinib in CLL patients included 94 patients with CLL/SLL, 22 of whom were treatment-naïve [101]. Common side effects of any grade were contusion (35.1%), upper respiratory tract infection (33%), cough (25.5%), and diarrhea (21.3%). Neutropenia occurred in 6.4% as the most frequent grade 3/4 AE. At a median follow-up of 13.7 months, an ORR of 96.2% (95% CI, 89.2–99.2) and an estimated PFS at 12 months of 100% were reported. A phase 2, single-arm, multicenter study evaluating zanubrutinib in 91 patients with r/r CLL/SLL yielded similar results, with an ORR of 84.6% after a median follow-up of 15.1 months and an OS of 96% at 12 months [107]. The most common grade ≥3 adverse events were cytopenia and upper respiratory tract infections, which is in line with previous findings. Patients included showed high-risk characteristics, because 45% of patients had ≥2 lines of previous therapies, 24% had del(17p) or TP53 mutation, and 56% presented with unmutated IGHV status. In this trial, zanubrutinib seemed to be very effective in high-risk patients. Tam et al. recently reported results from a non-randomized cohort (Cohort C) of the phase 3 Sequoia trial (NCT03336333) including 109 treatment-naïve CLL patients with del(17p) who were treated with zanubrutinib monotherapy. The ORR was 94.5% and the estimated 18-month PFS rate was 88.6%. The most common adverse events were respiratory infections and neutropenia. Atrial fibrillation and major bleedings occurred in three and six patients, respectively [108]. When combined with the CD20-antibody obinutuzumab, adverse events seem to occur more often but do not change in their nature; upper respiratory tract infections (51%) and neutropenia (44%) were still the most common [109]. Combination therapy of zanubrutinib and obinutuzumab resulted in an ORR of 100% for tn CLL patients and 92% for r/r CLL patients at a median follow-up of 29 months. A phase 2 trial tested the safety and efficacy of adding venetoclax to this combination in 39 patients with tn CLL [110]. The endpoints assessed were uMRD in peripheral blood (PB) and bone marrow (BM). The combination of obinutuzumab, venetoclax, and zanubrutinib was well tolerated, with no new safety concerns, and achieved uMRD in 68% and 51% in PB and BM, respectively.

## 8. LOXO-305

Despite the success of BTKis, acquired and primary resistance to conventional (covalent) BTKis can confer clinical resistance [111]. LOXO-305 (Loxo Oncology, Stamford, CT, USA) is a selective, reversible, non-covalent BTKi which showed potent BTK inhibition regardless of the presence of a C481S mutation in preclinical models [112]. Recently, results from an early phase 1/2 trial investigating oral LOXO-305 in patients with r/r CLL/SLL and non-Hodgkin lymphoma were published by Mato and colleagues.

They enrolled 323 patients in a first-in-human, multicenter, open-label, phase 1/2 trial to evaluate the safety and efficacy of oral pirtobrutinib (working name; formerly known as LOXO-305) in previously treated patients with B cell malignancies [113].

In this trial, patients were treated with pirtobrutinib across seven dose levels (25 mg, 50 mg, 100 mg, 150 mg, 200 mg, 250 mg, and 300 mg once per day). They observed no dose-limiting toxicities, leading to the phase 2 dose of 200 mg daily. Regarding safety profile, the most often seen adverse events were fatigue (20%), diarrhea (17%), and contusion (13%). Interestingly, grade 3 atrial fibrillation or flutter were not observed. Five (1%) patients discontinued treatment due to a treatment-related adverse event. All patients with r/r CLL or SLL were treated previously with a covalent BTKi with a median of four preceding lines of treatment. The ORR in CLL patients was 62% and did not differ between CLL patients with resistance to covalent BTKi resistance (67%) and patients with BTKi intolerance (52%) [113]. In summary, these early data show that pirtobrutinib is safe and active in patients with r/r CLL, including patients previously treated with covalent BTKis. However, the role of new, non-covalent BTK inhibitors in the treatment landscape of CLL will have to be clarified in upcoming trials.

## 9. Combination of BTKi and BCL2i

Ongoing trials are currently investigating the combination of BTKi with BCL2i, based on the idea of clinically complimentary activity due to the fact that BTKis are highly active in treating and shrinking nodal disease, while BCL2i is highly effective at clearing bone marrow and peripheral blood of CLL. Early stage trials showed promising results in both first-line [114] and relapsed/refractory [115] treatment settings. In these two trials, the treatment combination of BTKi (ibrutinib) and BCL2i (venetoclax) resulted in a high uMRD rate (36% in r/r CLL patients [115] and 61% in untreated high-risk and older patients with CLL [114]). However, it is still not clear what role CD20 monoclonal antibodies can play in this combination. An answer to that might be given in the recruiting ACE-CL-311 trial (NCT03836261), which will investigate the combination of venetoclax plus acalabrutinib with or without obinutuzumab compared to chemoimmunotherapy in patients with previously untreated CLL.

## 10. CAR-T Cell Therapy

In recent years, a new treatment option arose for CD19^+^ B cell malignancies: chimeric antigen receptor-engineered (CAR)-T cell immunotherapy [116]. Multiple clinical trials of CD19 directed CAR-T cell therapy have shown efficacy [117,118,119], which led to the approval of this therapy by the EMA and FDA for specific indications [118,120,121]. For the treatment of CLL, CAR-T cells are currently not FDA/EMA-approved. However, CAR-T cell therapy is available in clinical trials (NCT03331198; NCT03624036) and might be a very promising treatment option in r/r CLL, offering durable remissions with a manageable toxicity profile. In a pivotal trial, Porter et al. reported an ORR of 57% in a heavily pretreated CLL patient cohort (median of five previous therapies) with four complete remissions (4/14) and four partial remissions (4/14), underlining the strength of this therapeutic approach [122]. Moreover, uMRD was reached in patients who achieved CR, which led the authors to the suggestion that disease eradication with CAR-T cells is possible even in advanced CLL. Moving on, another phase 1/2 open-label clinical trial by Turtle and colleagues demonstrated that CD19 CAR-T cells are highly effective even in high-risk patients after BTKi treatment failure [123]. In this trial, the ORR was 74%, with a CR rate of 21% (4/19) and PR rate of 53% (10/19). Regarding uMRD, no disease was detectable by flow cytometry after CAR-T cell infusion in 88% of patients with marrow disease before CAR-T cell treatment. In 12 patients, deep IGH sequencing was performed. In seven patients (58%), CLL IGH sequences were not detectable after therapy in the bone marrow, resulting in a 100% PFS and OS (median follow-up 6.6 months) of these patients [123]. More recently, the open-label phase 1/2 trial TRANSCEND-CLL-004 was updated. In detail, results of 23 patients (22 were evaluable for efficacy) showed an ORR of 82% and a CR rate of 45% after a median follow-up of 9 months. In this trial, most patients (83%) were defined as high-risk, with a median of five prior therapies including BTKi therapy [124]. However, 60% of evaluable patients had uMRD in the bone marrow by day 30. Cytokine release syndrome (CRS) was reported in 74% (9% were grade 3–4) and neurotoxicity in 39% (22% were grade 3–4) of the patients, respectively. Due to more experience over recent years (e.g., using tocilizumab or corticosteroids), CAR-T cell-related toxic side effects became more manageable in daily clinical practice. Therefore, with the possible exception of allogeneic stem cell transplantation, these remarkable results might break the paradigm that CLL is not a curable disease.

## 11. Suggested Treatment Algorithm Upfront and at Relapse

Due to the tremendous changes over the last few years in the treatment landscape for CLL, the choice of treatment should be based on patient parameters and disease factors. Patient-related factors should include comorbidities (e.g., based on the Cumulative Illness Rating Score [125], go–go / fit vs. slow go/frail), organ function, and drug interaction. The disease factors are known genetic biomarkers/risk factors such as del(17p)/TP53 mutation, IGVH mutation status, and karyotype. In addition, patients should be enrolled in clinical trials whenever possible. These factors have to be considered and weighed in the process of providing individualized therapy for CLL patients.

## 12. Upfront Treatment

**Figure 1:**
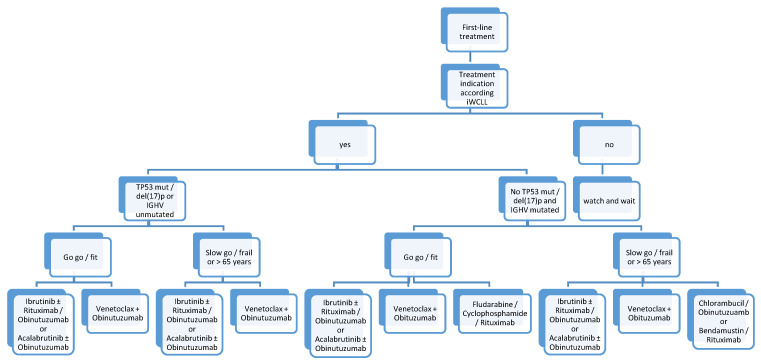
Frontline treatment algorithm in CLL.

Based on recently published randomized trials comparing CIT regimens with BTKi, namely, ibrutinib or acalabrutinib, BTKis are now considered a feasible option for all patients in first-line setting (see Table 1 and Figure 1). Therefore, comorbidities such as arterial hypertension or renal impairment, need for oral anticoagulation, and patient’s preference (unlimited therapy with BTKi or fixed treatment duration), should be taken into account when choosing first-line therapy in CLL patients. Until now, no head-to-head trial has shown superiority for one or another novel agent (BTKis or venetoclax). Therefore, venetoclax + obinutuzumab might be the combination of choice if a patient prefers a fixed treatment duration or if a patient suffers from cardiovascular comorbidities (hypertension and atrial fibrillation), or if there is a high risk of bleeding. In patients with high tumor burden and significant risk of tumor lysis syndrome, BTKis might be the treatment of choice, especially because of easier treatment initiation (no ramp-up needed, oral treatment). They also may be preferred when quick disease control is needed.

Nevertheless, even though novel agents might be the standard of care, CIT (chlorambucil/obinutuzumab, bendamustine/rituximab or FCR) is still an available treatment option in tn IGHV-mutated CLL patients without del(17p), TP53 mutation, or complex karyotype.

## 13. Treatment in Relapsed/Refractory Patients

**Figure 2:**
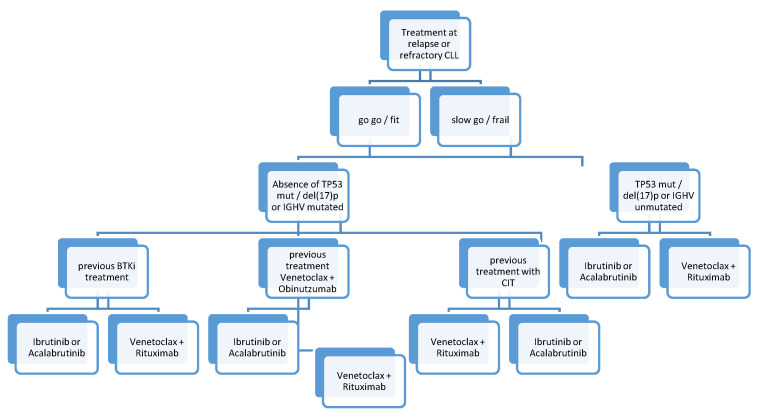
Treatment algorithm in relapsed/refractory CLL.

Similar to first-line therapy, r/r CLL should only be treated if symptomatic according to the International Workshop Group on CLL (iwCLL) criteria [4]. As with tn nCLL patients, novel therapeutics are superior to CIT regimens, leading to significantly better survival in r/r CLL patients [4,127]. Both combination treatment with venetoclax + rituximab and BTKis are feasible treatment options in r/r CLL patients (Figure 2 and Table 2).

We suggest that re-treatment with venetoclax + rituximab might also be a feasible treatment option if patients had a long duration of response without disease progression after first-line treatment with venetoclax + obinutuzumab (e.g., after 53.9 months, which is the median PFS in the MURANO trial [66]).

## 14. Conclusions

Within the last few decades, numerous advances in the treatment of CLL have been made, including the introduction of novel classes of targeted small molecules such as BCL2 inhibitors, BTK inhibitors, or PI3K inhibitors. These therapeutics have fundamentally changed the treatment of CLL and improved patient outcomes. An improved progression-free survival with these therapeutics over CIT in both settings, first-line and relapsed/refractory, was demonstrated in many randomized clinical trials. Of note, these chemotherapy-free regimens especially helped to improve the outcome in subgroups with previously very poor prognoses. Unanswered questions, such as whether two- or three-drug regimens are the best treatment choice or if an endless treatment might be better than a time-limited treatment, will hopefully be answered in the near future. Ongoing trials such as the recruiting CLL17 trial (NCT03701282) are addressing this issue.

Nevertheless, despite all these advances, CLL remains an incurable disease, necessitating new clinical trials to improve CLL treatment.

## Figures and Tables

**Table 1:** Overview of phase 3 trials in tn CLL.

Trial	Treatment Arms	ORR	OS	PFS	uMRD
ALLIANCE (A041202) Study [47]	Ibr vs. Ibr-R vs. BR	81% (BR) vs. 93% (Ibr) vs. 94% (Ibr-R)	2-year OS 95% (BR) vs. 90% (Ibr) vs. 94% (Ibr-R)	2-year PFS 74% (BR) vs. 87% (Ibr) vs. 88% (Ibr-R)	8% (BR) vs. 1% (Ibr) vs. 4% (Ibr-R)
E1912 study [74]	FCR vs. Ibr-R	96% (Ibr-R) vs. 81% (FCR)	3-year OS 92% (FCR) vs. 99% (Ibr-R)	3-year PFS 73% (FCR) vs. 89% (Ibr-R)	8% (Ibr-R) vs. 59% (FCR)
RESONATE-2 [19,54]	Ibr vs. Clb	86% (Ibr) vs. 35% (Clb)	5-year OS 83% (Ibr) vs. 68% (Clb)	5-year PFS 70% (Ibr) vs. 12% (Clb)	N.A.
iLLUMINATE study [76]	G-Ibr vs. G-Clb	88% (G-Ibr) vs. 73% (G-Clb)	Estimated 30-month OS 86% (G-Ibr) vs. 85% (G-Clb)	Estimated 30-month PFS 79% (G-Ibr) vs. 31% (G-Clb)	35% (G-Ibr) vs. 25% (G-Clb)
ELEVATE TN [82]	Acb vs. G-Acb vs. G-Clb	94% (G-Acb) vs. 79% (G-Clb) vs. 85% (Acb)	Estimated 24-month OS 95% (G-Acb) vs. 95% (Acb) vs. 92% (G-Clb)	Estimated 24-month PFS 93% (G-Acb) vs. 87% (Acb) vs. 47% (G-Clb)	N.A.
CLL14 trial [27,126]	Ven-G vs. G-Clb	85% (Ven-G) vs. 71% (G-Clb)	24-month OS 92% (Ven-G) vs. 93% (G-Clb)	3-year PFS 82% (Ven-G) vs. 50% (G-Clb)	76% (Ven-G) vs. 35% (G-Clb) 18 months after treatment 47% (Ven-G) vs. 7% (G-Clb)

Abbreviations: Ibr, ibrutinib; R, rituximab; F, fludarabine; C, cyclophosphamide; Clb, chlorambucil; G, obinutuzumab; Acb, acalabrutinib; Ven, venetoclax; N.A., not available.

**Table 2:** Overview of phase 3 trials in r/r-CLL.

Trial	Treatment Arms	ORR	OS	PFS	uMRD
ASCEND [84]	Acb vs. Investigators choice (Idl-R or BR)	81% (Acb) vs. 75%	12-months OS 94% (Acb) vs. 91%	Estimated 12-month PFS 88% (Acb) vs. 68% (Idl-R) or 69% (BR)	N.A.
MURANO [26,128]	Ven-R vs. BR	92% (Ven-R) vs. 72% (BR)	4-year OS 85% (Ven-R) vs. 67% (BR)	4-year PFS 57% (Ven-R) vs. 5% (BR)	N.A.
RESONATE-1 [18,129]	Ibr vs. Ofa	86% (Ibr) vs. 24% (Ofa)	3-year OS 74% (Ibr) vs. 65% (Ofa)	3-year PFS 59% (Ibr) vs. 3% (Ofa)	N.A.

Abbreviations: Acb, acalabrutinib; B, bendamustine; Ibr, ibrutinib; Idl, idelalisib; Ofa, ofatumumab; R, rituximab; Ven, venetoclax; N.A., not available.
